# Constant Temperature Approach for the Assessment of Injection Molding Parameter Influence on the Fatigue Behavior of Short Glass Fiber Reinforced Polyamide 6

**DOI:** 10.3390/polym13101569

**Published:** 2021-05-13

**Authors:** Selim Mrzljak, Alexander Delp, André Schlink, Jan-Christoph Zarges, Daniel Hülsbusch, Hans-Peter Heim, Frank Walther

**Affiliations:** 1 Department of Materials Test Engineering (WPT), TU Dortmund University, 44227 Dortmund, Germany; alexander.delp@tu-dortmund.de (A.D.); daniel.huelsbusch@tu-dortmund.de (D.H.); frank.walther@tu-dortmund.de (F.W.); 2 Institute of Material Engineering-Polymer Engineering, University of Kassel, 34125 Kassel, Germany; andre.schlink@uni-kassel.de (A.S.); zarges@uni-kassel.de (J.-C.Z.); heim@uni-kassel.de (H.-P.H.)

**Keywords:** fatigue testing, X-ray microtomography, short glass fiber, polyamide 6, injection molding, self-heating, testing methodology, digital volume correlation

## Abstract

Short glass fiber reinforced plastics (SGFRP) offer superior mechanical properties compared to polymers, while still also enabling almost unlimited geometric variations of components at large-scale production. PA6-GF30 represents one of the most used SGFRP for series components, but the impact of injection molding process parameters on the fatigue properties is still insufficiently investigated. In this study, various injection molding parameter configurations were investigated on PA6-GF30. To take the significant frequency dependency into account, tension–tension fatigue tests were performed using multiple amplitude tests, considering surface temperature-adjusted frequency to limit self-heating. The frequency adjustment leads to shorter testing durations as well as up to 20% higher lifetime under fatigue loading. A higher melt temperature and volume flow rate during injection molding lead to an increase of 16% regarding fatigue life. In situ X-ray microtomography analysis revealed that this result was attributed to a stronger fiber alignment with larger fiber lengths in the flow direction. Using digital volume correlation, differences of up to 100% in local strain values at the same stress level for different injection molding process parameters were identified. The results prove that the injection molding parameters have a high influence on the fatigue properties and thus offer a large optimization potential, e.g., with regard to the component design.

## 1. Introduction

Due to their lightweight potential combined with good mechanical properties, injection-molded short glass fiber reinforced plastics, hereinafter referred to as glass fiber reinforced composites (GFC), are being used in ever-increasing areas of application. GFC are now also implemented in components exposed to high loads, which were originally reserved for metallic materials. The largest market is the automotive industry, where fiber reinforced plastics are increasingly popular to achieve the required weight reduction and thus a reduction in emissions [1,2]. They are mainly used in paneling parts, but also covers, connector systems, tank systems, bodywork, and structural components [3].

The low weight, cost savings, and simple production of geometrically demanding components in large quantities are leading to a rapid increase in the use of short glass fiber reinforced thermoplastics in the processing and substitution of metallic components [4,5,6,7]. The use of glass fiber reinforced polyamide for, e.g., oil pans, bearings, and under-the-hood applications allows a weight reduction of up to 50% compared to a metallic series component [3]. By reinforcing the polyamide with glass fibers, an increase in stiffness and strength is achieved while at the same time reducing the creep tendency. The density of the fiber reinforced polyamide is only up to approx. 20% higher than that of the pure thermoplastic, preserving the excellent use for lightweight components.

Since the performance of such components can be a key aspect, the mechanical properties need to be known for the component design. GFC have been investigated for their static behavior in a large number of publications [8,9,10]. However, most GFC components are subjected to cyclic loads in their applications [5,6,11]. To achieve the same degree of reliability as with metallic components, it is necessary to carry out suitable tests to estimate the durability and investigate the fatigue behavior and underlying mechanisms. This becomes significantly more important because unforeseen material degradation can lead to catastrophic failure of components subjected to higher loads. In order to be able to predict the fatigue and failure properties using, e.g., a fatigue model [12,13], it is necessary to identify the properties of GFC under cyclic loading. In this context, the boundary conditions, e.g., the influence of injection molding parameters on the GFC fatigue performance and the testing method for fatigue behavior characterization, should not be neglected and have rarely been investigated. This is also due to the high amount of time required for fatigue testing. Therefore, a resource-and time-efficient testing method is necessarily required to investigate

(i) the influence of injection molding parameters
(ii) improve the reliability and comparability of the gathered information regarding the fatigue properties of GFC.

Short glass fiber reinforced thermoplastics, in particular, must be investigated with regard to (i) the influence of injection molding parameters. They show locally different microstructures and fiber orientations due to the processing influences, which have been extensively characterized by various methods [14,15,16,17]. These differences in the (micro)structure can have a significant influence on the mechanical properties and fatigue capability [6,18,19,20,21,22,23,24,25,26,27]. Therefore, a defined characterization of the process influences on the fatigue properties of the GFC is of great importance [18,28]. In various publications, the influence of fiber orientation in injection molded composites [20,25,29,30] as well as the influence of matrix material and different crystallization states on the fatigue properties have been characterized [24,31,32]. With regard to the process influences in injection molding, a significant influence of the melt and mold temperature as well as the volume flow on the dynamic strength and stiffness of the resulting GFC was demonstrated [15,33]. It becomes apparent that a detailed investigation regarding the influences of injection molding parameters on the fatigue properties of GFC is feasible with current testing methods only in a highly time-consuming manner for such a high number of parameters.

In addition to the deviating structures of the GFC due to injection molding parameters, the parameters of the cyclic loads also show an influence on the fatigue properties [17,25,34], whereby, among other aspects, different amplitudes were used [5] or tests were carried out in the range of very high cycle fatigue (VHCF) [25]. This demonstrates the need for a comparative testing method to (ii) improve the reliability and comparability of the gathered information regarding the fatigue properties of GFC. The dominant fatigue testing method factors influencing the performance of composites, including short fiber reinforced plastics, are applied load and testing frequency. One major reason is self-heating, which is why a separate consideration of load dependency and testing frequency (hereinafter referred to as frequency) dependency needs to be taken into account. The reason for self-heating is the thermoelastic effect, caused by material damping associated with viscoelastic dissipation and friction [35,36,37]. To identify the self-heating behavior, thermography plays a significant role as it is possible to detect the formation of a frequency and load-dependent temperature plateau after a comparative short testing duration (N >> 10^4^) at loads markedly below ultimate tensile strength (UTS) [34,35,38]. Due to a higher energy input, the self-heating increases with advancing load level [37,39]. In addition to specimen geometry [34], the frequency not only has a sensible influence on the self-heating but also regarding the lifespan of short fiber reinforced plastics. Regarding this, two opposing effects are considered: On one side, a high frequency leads to higher energy input and thereby to a rising self-heating until the melting or disintegration of matrix polymer material causes early failure. On the other side, a beneficial effect is gained through the avoidance of ratcheting, which is a great challenge at low frequency testing of polymers [36,38,40]. For an optimal consideration of both effects, a frequency-adjusted testing method based on the inducted energy rate has been proposed shortly. With this procedure, it is possible to shorten the testing duration by use of a thermal-applied optimized frequency for each load step while minimizing the occurring ratcheting at the necessarily low frequency for high load testing. As a result, a load step and load ratio-dependent limit frequency based on energy input is introduced in such a way that the self-heating remains constantly under ΔT < 2 K [37]. This enables a comparative characterization method while still reducing the overall testing duration; however, the feasibility of applying such an approach to short fiber reinforced plastics is still unknown.

For the characterization of the injection molding parameter and fatigue damage induced changes in the microstructure, the non-destructive radiographic examination by X-ray microtomography (µCT) is groundbreaking. Since failure occurs as a process of a sum of interaction between local damage spots [41], rather than a dominant crack, µCT enables a holistic analysis. The reliable qualitative and quantitative visualization of inner structures such as fibers, agglomerations, and voids enable expanded ways of damage characterization [42,43,44,45]. Further improvement is possible by extending µCT with in situ testing. By using this technique, it is possible to execute µCT recordings at applied load to identify damage mechanisms [46] without crack closure effects. In combination with fatigue testing, sequential or intermittent tests can be conducted, and the material state recorded to establish an understanding regarding damage initiation and propagation occurring in the material inner. With the use of digital volume correlation (DVC), deformation and strain in the material inner can be analyzed and correlated to the damage state recorded through µCT, extending the possibilities even further. In this regard, relaxation and creep of the material need to be taken into account, since µCT scans can take several hours and therefore lead to a blurred image and inaccurate representation of the material state [45,46].

In this study, short fiber reinforced polyamide 6 is investigated regarding the influence of injection molding parameters on the fatigue behavior using a constant temperature approach, which limits self-heating for comparable and reliable results. The three injection molding parameters melt temperature, mold temperature and volume flow rate were considered and varied to establish a comprehensive overview regarding their influence. Multiple frequency tests were used to determine the self-heating behavior of the investigated material, leading to a mathematical description of the temperature, testing frequency and fatigue load relationship. For the fatigue tests, metrology was used to measure temperature and strain development to conclude on the specimens’ self-heating and damage state using stress–strain hysteresis values. Changes in microstructure were determined using in situ µCT combined with digital volume correlation to conclude on the injection molding parameter-dependent fiber orientation and fiber length distribution, as well as the resulting local strain distribution after the fatigue load.

## 2. Materials and Methods

### 2.1. Materials

For the manufacturing of test specimens, polyamide 6 (PA6) with a glass fiber content of 30 wt.% (BASF, Ultramid B3EG6, Ludwigshafen am Rhein, Germany) was used. According to the manufacturer, this material for injection molding processing offers high mechanical strength, stiffness, and thermal stability at a density of 1.36 g/cm³ and is therefore particularly suitable for components and machine elements as well as high-grade electrical insulation. The melting temperature of the material *T_m_* = 220 °C and the melt volume rate (MVR) is 35 cm³ / 10 min (275 °C / 5 kg) according to ISO 1133. The glass fibers show a diameter of approx. *d_f_* = 12 µm and a mean glass fiber length of approx. *l_f_* = 400 µm.

### 2.2. Injection Molding

**Figure 1:**
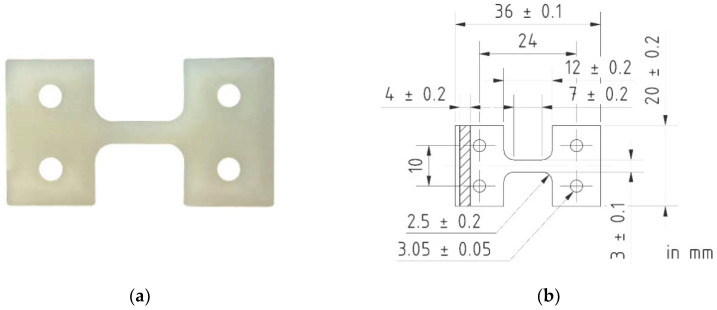
(**a**) Image of a tensile test specimen from Ultramid B3EG6 and (**b**) technical drawing with dimensioning.

For experimental investigations regarding the influence of injection molding parameters on the fiber distribution/alignment and corresponding mechanical properties of the composites, tensile test specimens were manufactured with the injection molding process. The specimens show a geometry that is suitable for the in situ stage of the X-ray µCT. An image of a representative specimen and its dimensions are shown in Figure 1.

Prior to the injection molding process, the glass fiber reinforced PA6 was dried using an air dryer (TORO-systems, TR-Dry-Jet EASY 15, Igensdorf, Germany) until a moisture content of approx. 0.2% was achieved. The test specimens were manufactured using a hydraulic injection molding machine (Arburg, Allrounder 320C Golden Edition, Loßburg, Germany), with a screw diameter of 25 mm and a clamping force of 500 kN.

Depending on the volume flow rate, the cycle time was approx. 45 s, including a packing time of 8.5 s and a cooling time of 30 s. The melt temperature, the volume flow rate, and the mold temperature showed the greatest influence of all injection molding parameters in previous tests and were varied in the experimental design. Considering the properties of the used material, the values of the injection molding parameter configurations can be seen in Table 1.

The packing pressure was constantly set to 400 bar. The feasibility of the extreme values of the considered injection molding parameters was characterized by filling and packing pressure studies in preliminary tests.

Each of the eight configurations of the injection molding parameters was used to manufacture ten process cycles with four specimens each. Prior to the production of the test specimens considered here, about 10 cycles of each parameter configuration were produced to stabilize the injection molding process and thus create constant processing conditions.

### 2.3. Tensile Testing

Quasi-static tensile tests were carried out at a speed of 5 mm/min according to EN ISO 527 using a universal testing machine (UPM 1446, F_max_ = ± 10 kN, Zwick Roell, Ulm, Germany). During the tests, stress–strain curves were recorded and Young’s modulus, the tensile strength, and the elongation at break were evaluated. The stroke strain values represent the strain derived from the measured change in stroke relative to the specimens’ measuring length of 7 mm (Figure 1b). Five specimens were tested for each parameter configuration of the experimental design.

### 2.4. Fatigue Testing

**Figure 2:**
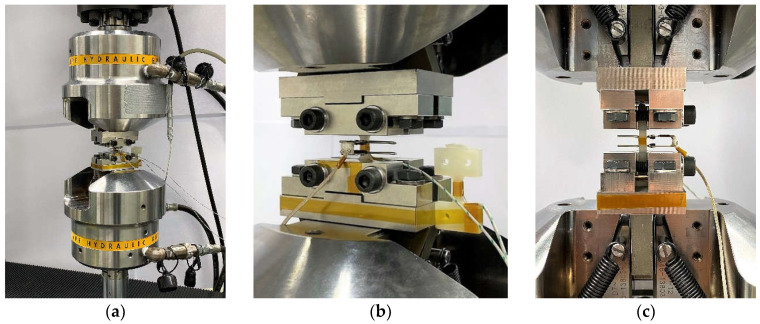
Mechanical testing setup: (**a**) servo-hydraulic testing frame with mounted custom CT specimen clamping adapter, (**b**) diagonal view of clamping adapter with mounted extensometer and thermocouples with reference specimen, (**c**) side view.

For fatigue testing, a servo-hydraulic testing system (EHF-EV50, F_max_ = ± 50 kN, Shimadzu, Kyoto, Japan) was used. Preliminary to the investigations of the mechanical properties, the polymer related self-heating effect, influenced by load level and frequency, was examined, since a reliable and comparative testing methodology needed to be established. Therefore, multiple frequency tests (MFT) at maximum stresses of σ_max_ = 30, 50, 70, 90 MPa with start frequencies (f_start_) and frequency increase steps (Δf) according to Table 2 for each maximum stress level were conducted with a sinusoidal load-time function under tension–tension loading (*R* = 0.1). Each frequency step was held for a time of *t* = 900 s to allow temperature stabilization. Tests were conducted up to a limited increase in surface temperature (ΔT_lim_) of 5 K, which enabled the description of the relationship between frequency and load level as a function of the desired maximum increase in surface temperature. Due to the required consistent high control accuracy of the testing machine, frequencies of 24 Hz were not exceeded. The temperature measurement was carried out with thermocouples type K, using one reference element on an unloaded specimen and one applied to the center specimen section as shown in Figure 2. Stress–strain hysteresis measurement for the detection of material degradation by the dynamic Young’s modulus (E_dyn_) was executed using a tactile extensometer (EXA 5–0.5, l_0_ = 5 mm ± 10%, Sandner-Messtechnik, Biebesheim, Germany). The desired maximum increase in surface temperature was set to 2 K to achieve a negligible impact of self-heating on the mechanical properties while attaining acceptable testing duration compared to a constant frequency of *f* = 5 Hz. Using multiple amplitude tests (MAT), the adjusted frequencies, which are reduced with increasing maximum stress, are compared to testing with constant frequency to demonstrate the significance of this aspect. In the MAT, the maximum starting stress (σ_max,start_) is set to 40 MPa and increased stepwise by Δσ_max_ = 2.5 MPa after every ΔN = 2500 cycles until failure.

MAT with such testing parameters were used further for examination of the fatigue behavior and differences in performance and damage development with respect to changing injection mold parameters.

### 2.5. X-ray Microtomography Analysis of the Composite Structure

In order to explain the deviations in the mechanical properties resulting from different injection molding parameter configurations based on the inner structures (fiber orientation and fiber length distribution), X-ray microtomography measurements were carried out. These were realized on specimens of parameter configurations 1 and 8 to cover the largest difference in properties.

**Figure 3:**
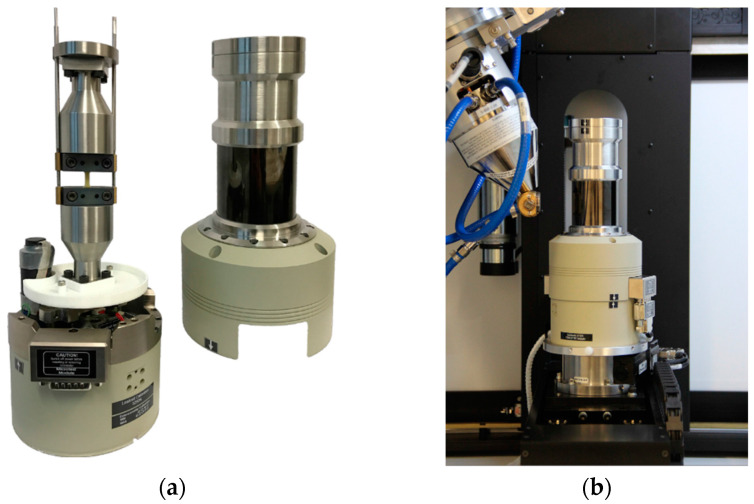
(**a**) In situ CT testing stage (**b**) implemented in CT chamber.

In situ µCT measurements were performed before testing and after defined MAT steps. This enables visualization and analysis of the injection molding parameter-dependent composition of the inner structures regarding fiber distribution, orientation, and agglomerations as well as digital volume correlation for deformation and strain measurement. This can be correlated to the recorded measurement data during the fatigue tests. These in situ µCT scans were recorded with a μCT (Nikon XT H 160, Nikon Corporation, Tokyo, Japan) in combination with an in situ CT testing stage (see Figure 3) (CT5000, Deben UK, Bury Saint Edmunds, UK), which was used to apply 90% of σ_max_ according to the MAT during the CT scan to overcome crack closure effects. CT acquisition parameters were defined as follows: tube voltage 60 kV, tube current 117 µA, exposure time 1000 ms, 1583 projections, 4-fold projection superimposition at a voxel size of 7 µm. The volumes were reconstructed using the software Nikon Inspect-X-XT4.4.2.

To enable a highly detailed analysis of the molding parameter-dependent fiber orientation and fiber length distribution, an additional characterization was conducted using an X-ray microscope (Zeiss Xradia Versa 520, Carl Zeiss, Oberkochen, Germany). The measurements were performed at a voltage of 80 kV and a current of 87 μA using the low energy filter LE2 and a magnification of 4x. A total of 1601 images were acquired with a voxel size of 2.64 μm, binning setting 1, and an exposure time of 8 s for each image. The single images were reconstructed using the Zeiss XMReconstructor software.

Further image processing was carried out utilizing a 3D data visualization and analysis software system and the XFiber extension (Avizo 9.4, Thermo Fisher Scientific, Waltham, US) for the quantitative analysis of fiber properties. Cylinder correlation was used to characterize the fiber orientation in the specimens. To segment the fibers, a tracing algorithm was applied to the resulting correlation lines. The detected fibers can be quantitatively evaluated with regard to their fiber length and orientation in the test specimen. XFiber extension settings for cylinder correlation and the tracing algorithm settings are shown in Table 3.

The fiber orientation is specified with the parameter Θ (theta), which describes the angle between the z-axis and the xy-plane and therefore indicates the degree of orientation in the flow direction. A value of Θ = 0° indicates an orientation in the flow direction and a value of Θ = 90° indicates an orientation transverse to the flow direction [47].

## 3. Results

### 3.1. Tensile Properties

**Figure 4:**
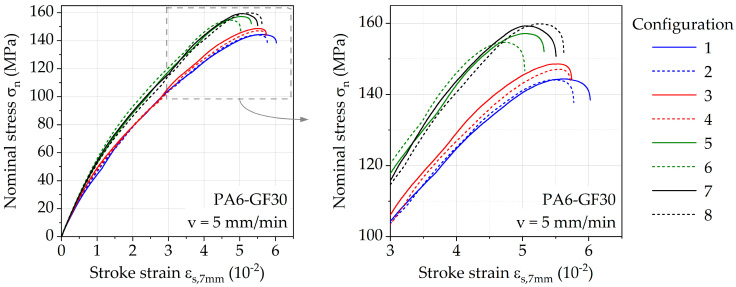
Exemplary stress–strain curves of PA6-GF30 for various injection molding parameter configurations.

Exemplary stress–strain curves for each investigated parameter configuration (Table 1) are shown in Figure 4. The curve colors differentiate between mold temperatures, while line types individually assign different volume flow rates. All curves follow a similar course, showing that the general tensile behavior of PA6-GF30 is not affected by the injection molding parameters, rather than the mechanical properties. The melt temperature seems to have the most impact, visible at the separation of the curves 1 to 4 (250 °C) and 5 to 8 (290 °C), resulting in the highest change in stiffness as well as tensile strength.

**Figure 5:**
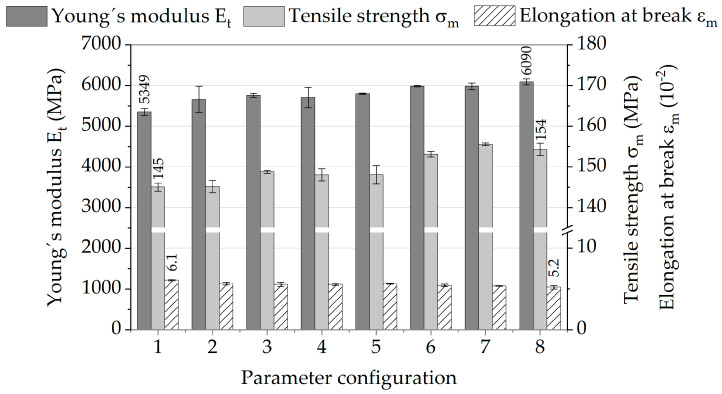
Quasi-static tensile properties of different injection molding parameter configurations.

To obtain an overall impression about the quantitative differences in mechanical properties, the mean values and standard deviations of Young’s modulus (E_t_), tensile strength (σ_m_), and elongation at break (ε_m_) resulting from all quasi-static tensile tests are shown in Figure 5. The results show that parameter configuration 1 has the lowest mean values of Young’s modulus and tensile strength but the highest values of elongation at break, while parameter configurations 7 and 8 show the highest mean values of Young’s modulus and tensile strength and the lowest elongations at break.

Based on the results of the quasi-static tests, it is obvious that especially a higher melt temperature (as seen in [33] for a PA6,6-based nanocomposite) and higher mold temperature (as shown by [31] for polycarbonate and [32] for polypropylene), but also a higher injection volume flow rate can lead to higher mechanical properties. It can be assumed that these effects will be more evident in the results of the fatigue tests.

### 3.2. Self-Heating—Adjusted Frequency for Constant Change in Temperature

**Figure 6:**
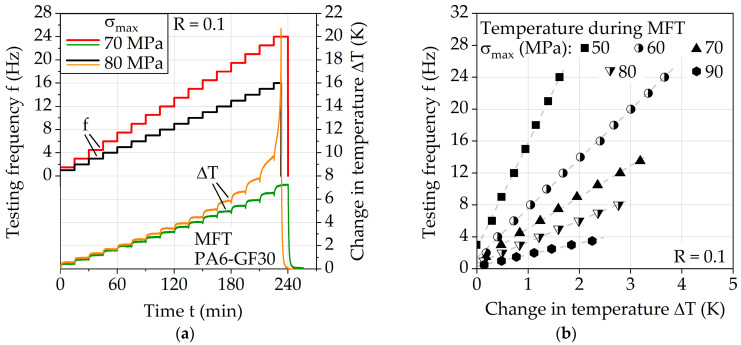
(**a**) Results of multiple frequency test at σ_max_ = 70 and 80 MPa; (**b**) linear temperature–testing frequency relationship visualized for σ_max_ = 50, 60, 70, 80 and 90 MPa.

Multiple frequency tests (MFT) were conducted using the parameters given in Table 2. Figure 6a exemplarily shows the frequency-dependent change in temperature measured during MFT at σ_max_ = 70 and 80 MPa. The used testing methodology enables the measurement of stable temperature plateaus during the fatigue load for a characterization of the relationship between frequency and change in the temperature of PA6-GF30. For 70 MPa, the change in temperature appears in constant increases with the establishment of temperature plateaus up until approx. *f* = 14 Hz. This is for 80 MPa only the case up until approx. *f* = 9 Hz. At higher frequencies, higher relative increases in temperature are visible, indicating more complex causes such as material degradation due to excessive strain rates. Therefore, only data acquired before these irregular changes in temperature were considered. Figure 6b visualizes this relationship of frequency and the change in temperature for σ_max_ = 50, 60, 70, 80, and 90 MPa, which appears to be linear for each investigated stress level. This relationship will be denoted in further discussion by the function f_frequency_.

**Figure 7:**
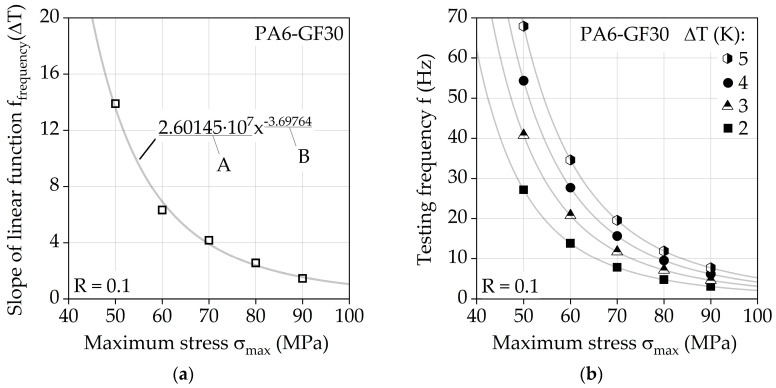
(**a**) Results of multiple frequency test at σ_max_ = 70 and 80 MPa; (**b**) linear temperature–testing frequency relationship visualized for σ_max_ = 50, 60, 70, 80 and 90 MPa.

Considering the linearity of f_frequency_ for each investigated stress level, a function can be described which approximately represents the slope of f_frequency_ for each σ_max_ desired. Figure 7a shows a curve based on the determined slopes from the linear functions in Figure 6b, which describes the slope of f_frequency_ with regard to σ_max_ by using a power function. This power function can be utilized to describe a function for a defined limit frequency f_limit_, leading to Equation (1). This equation, considering the values A and B from Figure 7a, describes the resulting change in temperature of PA6-GF30 under fatigue load with a stress ratio of *R* = 0.1 at room temperature without additional cooling. It is valid for specimens with the geometry shown in Figure 1b. For different surface-to-volume ratios, this may change significantly with specific investigations.

(1) $$f_{limit}\;\left(\Delta T,\;\sigma_{max}\right)=\Delta T\cdot A\cdot {\left(\sigma_{max}\cdot MPa^{-1}\right)}^{B}$$

Using Equation (1), the relationship between the maximum stress and frequency can be described for defined changes in temperature, as shown by Figure 7b for ΔT = 2, 3, 4, and 5 K. Since the measured values in Figure 6b do not start in the origin at 0 K, the resulting curves in Figure 7b determined by Equation (1) lead to a conservative approximation of frequencies. However, this difference is considered negligible given the magnitude, which is justifiable when considering the multiple amplitude test (MAT) results shown below.

The calculated limit frequencies f_limit_ for σ_max_ from 40 to 100 MPa, in respect of a constant change in temperature of 2 K, are given in Table 4 and are hereinafter used for adjusted frequency f_ΔT = 2K_ in MAT. Additionally, the test durations of MAT with adjusted frequency are shown, illustrating the large differences in duration between different steps of the MAT due to the stress level-dependent frequency changes. Considering a conventional testing method with a constant frequency of 5 Hz, significant time savings can be achieved. Comparing MAT with a constant frequency of 5 Hz to adjusted frequency from Table 4 up to σ_max_ = 90 MPa (2500 cycles per step), adjusted frequency results in a reduction in test duration by approx. 37%. Therefore, the result of the constant temperature approach is not just a temperature-controlled fatigue test, but rather a more efficient testing methodology itself.

### 3.3. Fatigue Properties

**Figure 8:**
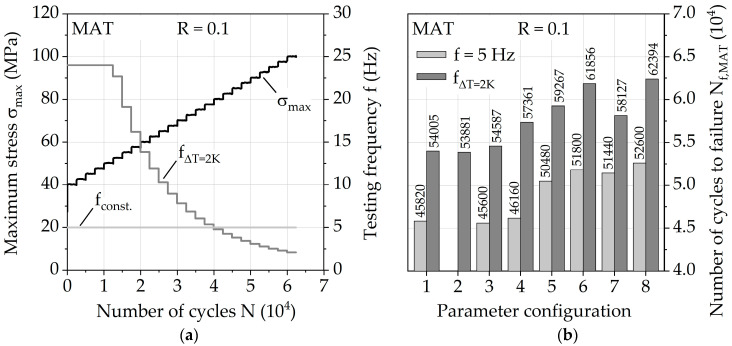
(**a**) Multiple amplitude test parameters visualized with constant testing frequency and adjusted frequency for a constant change in temperature ΔT = 2 K; (**b**) according numbers of cycles to failure N_f,MAT_ for all injection molding parameter configurations.

The fatigue behavior was investigated for the injection molding parameter configurations via a constant frequency f of 5 Hz and adjusted frequency f_ΔT = 2K_ for a change in temperature of ΔT = 2 K. Figure 8a visualizes the different frequencies for each MAT step during the test. The numbers of cycles to failure N_f,MAT_ with regard to MAT (Figure 8b) give an impression about the fatigue capability of the injection molding parameters since differences up to 15% are visible for *f* = 5 Hz and up to 16% for f_ΔT = 2K_. The results achieved by parameter configuration 8 show the highest values, representing a melt temperature of 290 °C, a mold temperature of 90 °C, and a volume flow rate of 28 cm^3^/s. Parameter configurations 1 and 2 (melt temperature = 250 °C, mold temperature 60 °C) show the lowest values. Increasing the melt temperature increases the fatigue life the most with up to 16%, while still significant gains up to 7% can be achieved through increasing the volume flow rate. Since the impact of the volume flow rate does not seem to be significant for the investigated lower mold temperature of 60 °C and a melt temperature of 250 °C, the overall temperature achieved by the polymer in the mold plays an important role which needs to be investigated in the future more in depth. The relative difference between the performance of the investigated injection molding parameters stays nearly the same between the used frequencies *f* = 5 Hz and f_ΔT = 2K_, but the absolute difference between overall fatigue life during MAT varies up to 20%. This demonstrates how immense the influence of frequency is on PA6-GF30 and why it has to be taken into account when comparing results between different testing methodologies.

**Figure 9:**
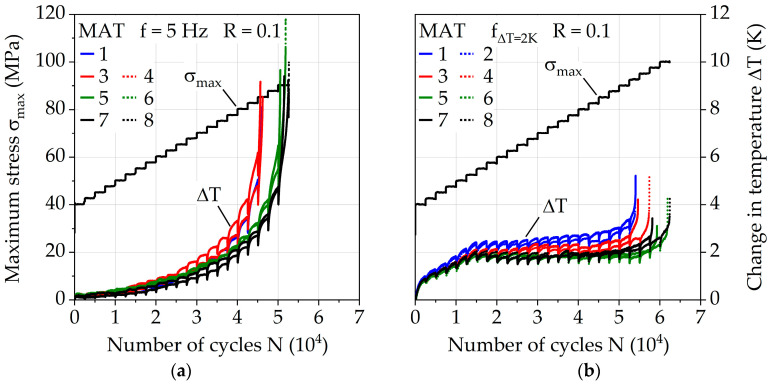
Change in temperature in multiple amplitude tests with (**a**) constant testing frequency of 5 Hz and (**b**) adjusted testing frequency for a constant change in temperature ΔT of 2 K.

The effectiveness of adjusted frequency for ΔT = 2 K is visible in Figure 9. With *f* = 5 Hz, exponential increases in the change in temperature over 10 K occur for each investigated parameter configuration. For f_ΔT = 2K_, the change in temperature is kept constant at around 2 K after σ_max_ = 52.5 MPa, where the adjusted frequency is fully applied for the first time in the MAT. Close to specimen failure, an increase in ΔT up to 5 K is visible, which is attributed to the damage but most likely does not significantly affect the number of cycles to failure.

**Figure 10:**
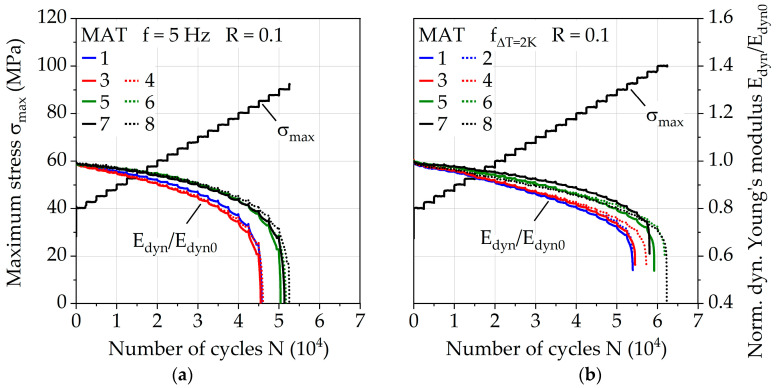
Normalized dynamic Young’s modulus in multiple amplitude tests with (**a**) constant testing frequency of 5 Hz and (**b**) adjusted testing frequency for a constant change in temperature ΔT of 2 K.

The measured dynamic Young’s modulus E_dyn_ (see Figure 10, plotted normalized using initial stiffness E_dyn0_) shows different curve progressions according to the temperature change of the investigated frequencies *f* = 5 Hz and f_ΔT = 2K_. Regarding the dynamic Young’s modulus E_dyn_, the influence of the melt temperatures of 250 and 290 °C is more evident, which could indicate an influence on the fiber orientation, which in turn significantly affects the stiffness of short glass fiber reinforced polymers. For f_ΔT = 2K_, an almost linear decrease in E_dyn_ is present, which changes to an exponential behavior shortly before failure. In the case of constant frequency, the decrease in E_dyn_ occurs far earlier (starting at around *N* = 3E4 cycles). Since the change in temperature until this point is below ΔT = 2 K, temperature-induced processes that result in a change of E_dyn_ are excluded.

**Figure 11:**
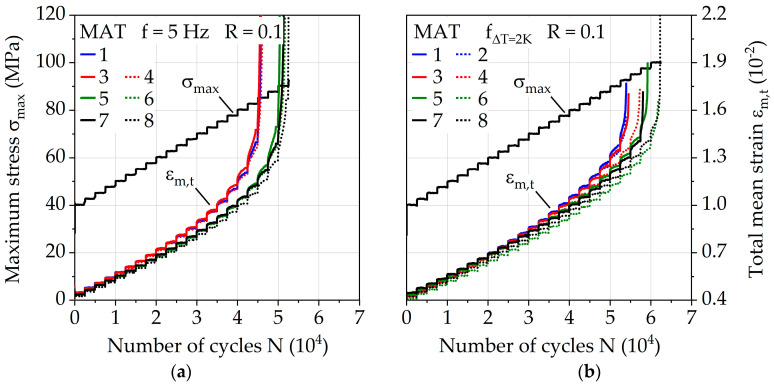
Total mean strain in multiple amplitude tests with (**a**) constant testing frequency of 5 Hz and (**b**) adjusted testing frequency for a constant change in temperature of ΔT = 2 K.

Besides the change in temperature, the test duration also needs to be considered when interpreting the mechanical properties of PA6-GF30. Due to the use of f_ΔT = 2K_, which leads to higher frequencies up to σ_max_ = 77.5 MPa of the MAT, the duration until the start of σ_max_ = 80 MPa is 3383 s, which is nearly 60% less compared to 8000 s when using the constant frequency of 5 Hz. This directly affects the creep behavior, which significantly affects lifetime as shown by [36,38,40]. The total mean strain ε_m,t_ can be used as an indicator for creep and is shown in Figure 11 for both investigated frequencies. The quantifiable as well as qualitative differences are visible, explaining the different progressions of E_dyn_ discussed earlier. With regard to the investigation of the influence of the injection molding parameters, especially for the parameter configurations 1 to 4, representing the melt temperature of 250 °C, creep seems to take place to a greater extent compared to the melt temperature of 290 °C. This affirms the assumptions made regarding the parameter configuration-dependent fiber orientation.

### 3.4. X-ray Microtomography Analysis of Composite Structure

X-ray microtomography images of the specimens were obtained to provide evidence for the suspected structural differences of the materials and their influence on the fatigue behavior of the specimens produced with different parameter configurations during injection molding. Due to the high differences in properties, configurations 1 and 8 were characterized here as those with the lowest and highest performance in the fatigue tests (see Figure 10 and Figure 11).

**Figure 12:**
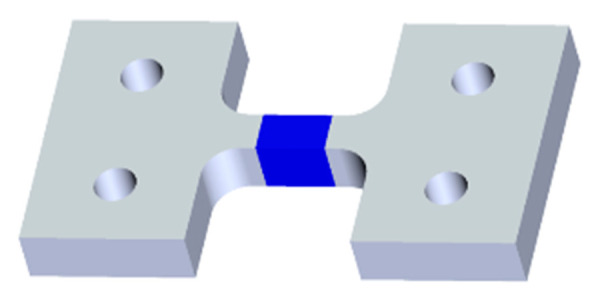
Area of the specimen characterized with X-ray microtomography (blue).

**Figure 13:**
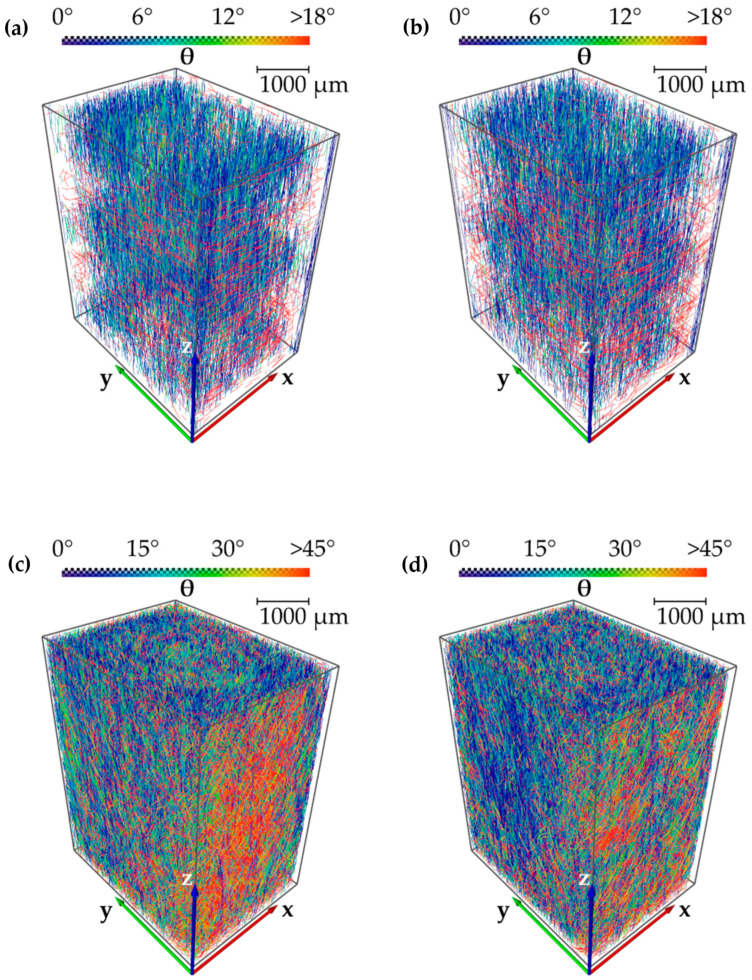
Results of fiber tracing from X-ray microtomography analysis and representation of angle Θ (theta) by a color scale in lower-resolution volumes (Nikon XT H 160) of configuration 1 (**a**) and 8 (**b**) and in high-resolution volumes (Zeiss Xradia Versa 520) of configuration 1 (**c**) and 8 (**d**).

For this purpose, the specimen areas, marked in blue in Figure 12, were first characterized with the X-ray microtomograph Nikon XT H 160, as described in Section 2.5, to determine differences in fiber length distribution and fiber orientation for configurations 1 and 8 with the results of the fiber tracing algorithm. However, the results of these microtomography images and the resulting detection and visualization of the fibers by the XFiber tracing algorithm (see Figure 13a,b) show that the resolution of the microtomography was not sufficient to adequately and representatively visualize the fibers.

For this reason, images were also generated on specimens of parameter configurations 1 and 8 using the X-ray microtomograph Zeiss Xradia Versa 520 with a significantly higher resolution (see Section 2.5), followed by an analysis using the XFiber tracing algorithm. It can be seen from Figure 13c,d that the µCT analysis with higher resolution led to a more precise detection of significantly more fibers and thus to much more meaningful analysis.

The fibers in the specimens detected by the XFiber tracing algorithm in the high-resolution X-ray microtomography images (Figure 13c,d) were subsequently used to calculate the fiber length distribution and the fiber orientation with angle Θ (theta) using the parameters listed in Section 2.5.

**Figure 14:**
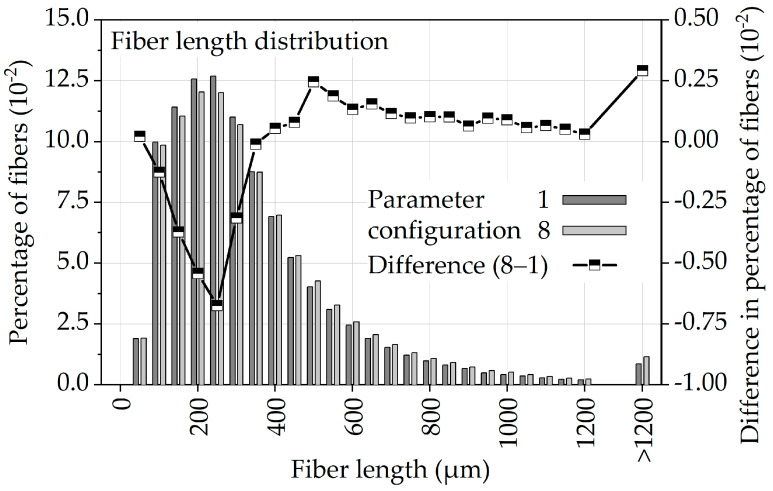
Normalized fiber length distribution of specimens with parameter configurations 1 and 8.

In Figure 14, the normalized fiber length distribution resulting from parameter configurations 1 and 8 is shown as a bar graph, while the line shows the difference between 1 and 8. From this difference, it can be seen that parameter configuration 8 shows fewer short fibers (< 350 µm) and more long fibers (> 350 µm) than parameter configuration 1. Based on the correlation of higher mechanical properties with higher fiber lengths [48,49,50,51,52], Figure 14 provides a first explanation for the improved fatigue properties of parameter configuration 8.

**Figure 15:**
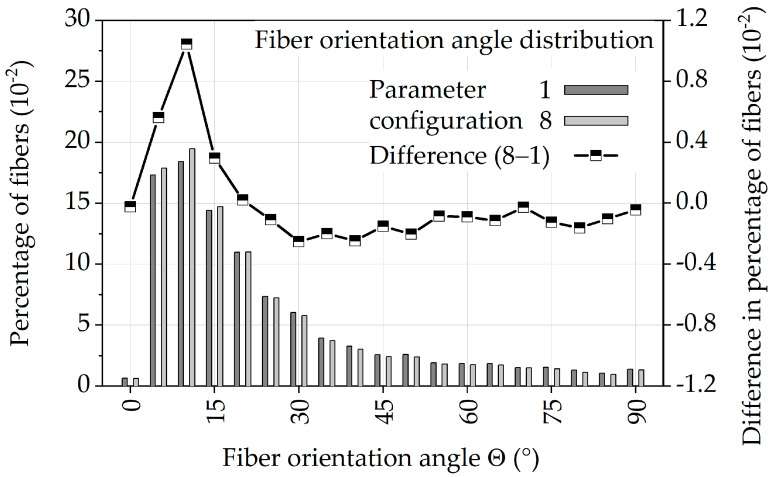
Normalized distribution of fiber orientation with angle Θ (theta) of specimens with parameter configurations 1 and 8.

Figure 15 shows the normalized distribution of fiber orientation for parameter configurations 1 and 8 with angle Θ (theta) as a bar graph and the difference of 1 and 8 as a line graph. From this, it can be seen that for both parameter configurations most of the fibers are present with angles Θ from 5 to 15°. The difference of the values shows that parameter configuration 8 has a larger number of fibers with angles Θ < 20° and fewer fibers with angles Θ > 20°. Thus, more fibers with an orientation closer to the loading direction (0°) are present in parameter configuration 8.

The correlation of higher mechanical properties with a fiber orientation in the loading direction [29,49,52,53,54,55,56,57] thus provides a further explanation for the better fatigue properties of parameter configuration 8 and confirms the assumptions about the process–structure–property correlation in Section 3.3.

### 3.5. Volume Correlation

**Figure 16:**
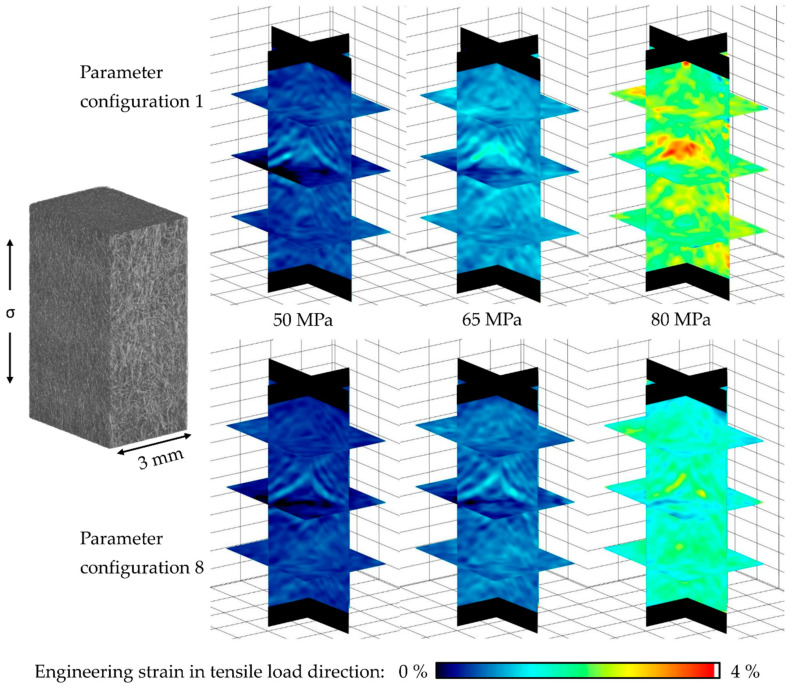
Strain distribution in tensile stress direction at selected σ_max_ of 50, 65 and 80 MPa for parameter configurations 1 and 8.

Using digital volume correlation (DVC) realized with the software DaVis 10.0.5.49808 (LaVision, Anna-Vandenhoeck-Ring 19, Göttingen, Germany), a visualization of the deformation and strain distribution within the material can be achieved, as it is shown in Figure 16 for the parameter configurations 1 and 8. The volumes were generated intermittently in MAT, using in situ µCT after the MAT steps σ_max_ = 50, 65, and 80 MPa, and represent the engineering strain in tensile load direction at each of the shown maximum stress levels. The significant differences in absolute strain values become apparent at first glance, showing that the lowest performing parameter configuration 1 has locally up to double the strain values compared to parameter configuration 8 at the same maximum stress level. In addition, a higher amount of concentrated localizations of higher strain accumulation can be determined for parameter configuration 1. This in general supports the findings from Section 3.4.

## 4. Conclusions

In this study, the influence of injection molding parameters on the fatigue behavior of short glass fiber reinforced polyamide 6 was investigated with regard to the process–structure–property correlations and to determine the achievable improvements in terms of, e.g., components with improved durability. To achieve comparative characterization results, an adjusted frequency approach for constant temperature realization was successfully applied. The following conclusions can be drawn:

With multiple frequency tests, the self-heating-dependent change in temperature of short glass fiber reinforced polyamide 6 can be determined sufficiently to describe the relationship between frequency and change in temperature. The result of the applied methodology is a power function, which describes the limit frequency for a defined change in temperature under fatigue load, considering a stress ratio of *R* = 0.1 at room temperature without additional cooling. Further, the test duration is reduced, since significantly higher frequencies at lower stresses lead to time saving.

The use of a constant frequency proved to be insufficient to determine the mechanical properties regarding the fatigue behavior, compared to the constant change in temperature. All tested configurations of injection molding parameters showed a high dependency on the frequency, leading to, e.g., up to 20% higher fatigue life in multiple amplitude tests when applying adjusted frequency for a material-dependent suitable constant temperature.

The significance of optimized injection molding parameters should not be underestimated. With a short time testing procedure using multiple amplitude tests, the differences in mechanical properties during fatigue load were characterized, showing that not just the fatigue life is affected, but also key properties such as mean strain and stiffness. The melt temperature showed the highest positive influence on fatigue performance, indicating that melt temperatures need to be chosen high enough to achieve an optimized morphology in terms of the resulting mechanical properties. The volume flow rate is the second parameter having a distinguishable positive impact on fatigue performance, pointing at the beneficial use of higher volume flow rates to distribute fibers in the material along the flow direction. Compared to both, the impact of the investigated mold temperatures seems to be negligible.

Analyzing the fiber length distribution and fiber orientation angle distribution between different injection molding parameters contributes substantially to the understanding of differences in mechanical properties. Comparing the worst with the best parameter combination regarding fatigue performance showed higher amounts of longer fibers as well as more fibers oriented in an angle smaller than 15° to the flow and load direction in the better performing specimen. Digital volume correlation was used to investigate the strain distribution in the volume inner of the material. The images illustrate injection parameter-dependent varieties in the amount of strain and local strain distribution, supporting the general observations made by fiber length and orientation analysis.

In future work, the injection molding parameter-dependent fatigue performance needs to be investigated thoroughly with an emphasis on further parameter variations and polymers. Even smaller degrees of variations should be considered to establish a well-founded data basis for setting up a representative model. This model could help to evaluate specific changes in parameters for the most efficient and best performing short fiber reinforced plastics, leading to improved quality of parts and/or savings of material due to constructive part optimizations because of the improved mechanical properties. The combination of conventional fatigue testing methods with non-destructive testing equipment such as X-ray microtomography is crucial for an in-depth understanding and, combined with digital volume correlation, is a game changer. Therefore, particular attention needs to be given to the intersection between those two for a direct transferability between microstructural properties and mechanical performance. By applying machine learning methods, an in-depth analysis would enable a comprehensive view of all influencing factors.

## Figures and Tables

**Table 1:** Injection molding parameter configurations.

Configuration [–]	Melt Temperature [°C]	Mold Temperature [°C]	Volume Flow Rate [cm³/s]
1	250	60	20
2	250	60	28
3	250	90	20
4	250	90	28
5	290	60	20
6	290	60	28
7	290	90	20
8	290	90	28

**Table 2:** MFT parameters for self-heating investigation.

**σ_max_**	50 MPa	60 MPa	70 MPa	80 MPa	90 MPa
**f_start_**	3 Hz	2 Hz	1.5 Hz	1 Hz	0.5 Hz
**Δ** **f**	3 Hz	2 Hz	1.5 Hz	1 Hz	0.5 Hz

**Table 3:** XFiber extension settings (Avizo 9.4) depending on the origin of μCT 3D data (Nikon/Zeiss microtomograph).

X-ray Microtomograph		Zeiss Xradia Versa 520	Nikon XT H 160
Cylinder length	[μm]	38	100
Angular sampling		5	5
Mask cylinder radius	[μm]	10	10
Outer cylinder radius	[μm]	6	6
Minimum seed correlation		200	150/144
Minimum continuation quality		80	80
Direction coefficient		0.2	0.2
Minimum distance	[μm]	10	12
Minimum length	[μm]	38	100

**Table 4:** Calculated frequencies for fatigue loads at given σ_max_ with ΔT up to 2 K according to Equation (1). Test duration shown for multiple amplitude test steps with length of 2500 cycles, individually and accumulated over test steps.

**σ_max_**	[MPa]	40	42.5	45	47.5	50	52.5	55	57.5	60	62.5	65	67.5	70
**f_ΔT = 2K_**	[Hz]	24	24	24	24	24	22.7	19.1	16.2	13.8	11.9	10.3	9	7.8
**t_N = 2500_**	[s]	104	104	104	104	104	110	131	154	181	210	243	278	321
**t_accumul._**	[s]	104	208	312	416	520	630	761	915	1096	1306	1549	1827	2148
**σ_max_**	[MPa]	72.5	75	77.5	80	82.5	85	87.5	90	92.5	95	97.5	100	
**f_ΔT = 2K_**	[Hz]	6.9	6.1	5.4	4.8	4.3	3.8	3.4	3.1	2.8	2.5	2.3	2.1	
**t_N = 2500_**	[s]	362	410	463	521	581	658	735	806	893	1000	1087	1190	
**t_accumul._**	[s]	2510	2920	3383	3904	4485	5143	5878	6684	7577	8577	9664	10,584	

## Data Availability

Not applicable.

## References

[1] Erden S., Ho K., Seydibeyoğlu M., Mohanty A., Misra M. (2017). Fiber reinforced composites. Fiber Technology for Fiber-Reinforced Composites.

[2] Schoßig M. (2011). Schädigungsmechanismen in Faserverstärkten Kunststoffen: Quasistatische und Dynamische Untersuchungen.

[3] Schürmann H. (2005). Konstruieren mit Faser-Kunststoff-Verbunden.

[4] Capela C., Oliveira S., Ferreira J. (2019). Fatigue behavior of short carbon fiber reinforced epoxy composites. Compos. Part B Eng..

[5] Eftekhari M., Fatemi A. (2017). Variable amplitude fatigue behavior of neat and short glass fiber reinforced thermoplastics. Int. J. Fatigue.

[6] Launay A., Marco Y., Maitournam M., Raoult I., Szmytka F. (2010). Cyclic behavior of short glass fiber reinforced polyamide for fatigue life prediction of automotive components. Procedia Eng..

[7] Mortazavian S., Fatemi A. (2017). Fatigue of short fiber thermoplastic composites: A review of recent experimental results and analysis. Int. J. Fatigue.

[8] Ferreira J.A.M., Costa J.D.M., Reis P.N.B. (1999). Static and fatigue behaviour of glass-fibre-reinforced polypropylene composites. Theor. Appl. Fract. Mech..

[9] Fu S.-Y., Lauke B., Mäder E., Yue C.-Y., Hu X. (2000). Tensile properties of short-glass-fiber- and short-carbon-fiber-reinforced polypropylene composites. Compos. Part A Appl. Sci. Manuf..

[10] Harris B. (2003). A historical review of the fatigue behaviour of fibre-reinforced plastics. Fatigue in Composites.

[11] Klimkeit B., Castagnet S., Nadot Y., El Habib A., Benoit G., Bergamo S., Dumas C., Achard S. (2011). Fatigue damage mechanisms in short fiber reinforced PBT+PET GF 30. Mater. Sci. Eng. A.

[12] Nouri H., Meraghni F., Lory P. (2009). Fatigue damage model for injection-molded short glass fibre reinforced thermoplastics. Int. J. Fatigue.

[13] Rolland H., Saintier N., Lenoir N., King A., Robert G. (2016). Fatigue mechanisms description in short glass fibre reinforced thermoplastic by microtomographic observations. Procedia Struct. Integr..

[14] Hessman P.A., Riedel T., Welschinger F., Hornberger K., Böhlke T. (2019). Microstructural analysis of short glass fiber reinforced thermoplastics based on X-ray micro-computed tomography. Compos. Sci. Technol..

[15] Lafranche E., Krawczak P., Ciolczyk J.P., Maugey J. (2005). Injection moulding of long glass fiber reinforced polyamide 66: Processing conditions/microstructure/flexural properties relationship. Adv. Polym. Technol. J. Polym. Process. Inst..

[16] Vincent M., Giroud T., Clarke A.R., Eberhardt C.N. (2005). Description and modeling of fiber orientation in injection molding of fiber reinforced thermoplastics. Polymers.

[17] Raphael I., Saintier N., Robert G., Béga J., Laiarinandrasana L. (2019). On the role of the spherulitic microstructure in fatigue damage of pure polymer and glass-fiber reinforced semi-crystalline polyamide 6. Int. J. Fatigue.

[18] SadAbadi H., Ghasemi M. (2007). Effects of Some Injection Molding Process Parameters on Fiber Orientation Tensor of Short Glass Fiber Polystyrene Composites (SGF/PS). J. Reinf. Plast. Compos..

[19] Mortazavian S., Fatemi A. (2015). Fatigue behavior and modeling of short fiber reinforced polymer composites including anisotropy and temperature effects. Int. J. Fatigue.

[20] Song J.H., Lim J.K. (2003). Low cycle fatigue of pps polymer injection welds (II)—Fiber orientation and fracture mechanism. KSME Int. J..

[21] Wilmes A., Hornberger K. (2015). Influence of Fiber Orientation and Multiaxiality on the Fatigue Strength of Unnotched Specimens—Lifetime Estimation. Procedia Eng..

[22] Santharam P., Marco Y., Le Saux V., Le Saux M., Robert G., Raoult I., Charrier P. (2020). Fatigue criteria for short fiber-reinforced thermoplastic validated over various fiber orientations, load ratios and environmental conditions. Int. J. Fatigue.

[23] Abdo D., Gleadall A., Silberschmidt V.V. (2019). Damage and damping of short-glass-fibre-reinforced PBT composites under dynamic conditions: Effect of matrix behaviour. Compos. Struct..

[24] Fouchier N., Nadot-Martin C., Conrado E., Bernasconi A., Castagnet S. (2019). Fatigue life assessment of a Short Fibre Reinforced Thermoplastic at high temperature using a Through Process Modelling in a viscoelastic framework. Int. J. Fatigue.

[25] Lee C.S., Kim H.J., Amanov A., Choo J.H., Kim Y.K., Cho I.S. (2019). Investigation on very high cycle fatigue of PA66-GF30 GFRP based on fiber orientation. Compos. Sci. Technol..

[26] Fatemi A., Mortazavian S., Khosrovaneh A. (2015). Fatigue behavior and predictive modeling of short fiber thermoplastic composites. Procedia Eng..

[27] De Monte M., Moosbrugger E., Quaresimin M. (2010). Influence of temperature and thickness on the off-axis behaviour of short glass fibre reinforced polyamide 6.6—Cyclic loading. Compos. Part A Appl. Sci. Manuf..

[28] Zhou Y., Mallick P. (2005). Effects of melt temperature and hold pressure on the tensile and fatigue properties of an injection molded talc-filled polypropylene. Polym. Eng. Sci..

[29] Bernasconi A., Davoli P., Basile A., Filippi A. (2007). Effect of fibre orientation on the fatigue behaviour of a short glass fibre reinforced polyamide-6. Int. J. Fatigue.

[30] Cosmi F., Bernasconi A. (2010). Fatigue Behaviour of Short Fibre Reinforced Polyamide: Morphological and Numerical Analysis of Fibre Orientation Effects.

[31] Dar U.A., Xu Y.J., Zakir S.M., Saeed M.-U. (2016). The effect of injection molding process parameters on mechanical and fracture behavior of polycarbonate polymer. J. Appl. Polym. Sci..

[32] Farotti E., Natalini M. (2018). Injection molding. Influence of process parameters on mechanical properties of polypropylene polymer. A first study. Procedia Struct. Integr..

[33] Doagou-Rad S., Islam A., Jensen J. (2017). Influence of Processing Conditions on the Mechanical Behavior of MWCNT Reinforced Thermoplastic Nanocomposites. Procedia CIRP.

[34] Jegou L., Marco Y., Le Saux V., Calloch S. (2013). Fast prediction of the Wöhler curve from heat build-up measurements on Short Fiber Reinforced Plastic. Int. J. Fatigue.

[35] La Rosa G., Risitano A. (2000). Thermographi methodology for rapid determination of the fatigue limit of materials and mechanical components. Int. J. Fatigue.

[36] Eftekhari M., Fatemi A. (2016). On the strengthening effect of increasing cycling frequency on fatigue behavior of some polymers and their composites: Experiments and modeling. Int. J. Fatigue.

[37] Hülsbusch D., Kohl A., Striemann P., Niedermeier M., Strauch J., Walther F. (2020). Development of an energy-based approach for optimized frequency selection for fatigue testing on polymers—Exemplified on polyamide. Polym. Test..

[38] Zhou Y., Mallick P.K. (2006). Fatigue performance of an injection-molded short E-glass fiber-reinforced polyamide 6, I. Effects of orientation, holes, and weld line. Polym. Compos..

[39] Muller L., Roche J.-M., Hurmane A., Peyrac C., Laurent G. Experimental monitoring of the self-heating properties of thermoplastic composite materials during tensile and cyclic tests. Proceedings of the 12th International Fatigue Congress (FATIGUE 2018).

[40] Bernasconi A., Kulin R.M. (2009). Effect of frequency upon fatigue strength of a short glass fiber reinforced polyamide 6: A superpo-sition method based on cyclic creep parameters. Polym. Compos..

[41] Reifsnider K., Raihan M.R., Vadlamudi V. (2016). Heterogeneous fracture mechanics for multi-defect analysis. Compos. Struct..

[42] Schilling P.J., Karedla B.R., Tatiparthi A.K., Verges M.A., Herrington P.D. (2005). X-ray computed microtomography of internal damage in fiber reinforced polymer matrix composites. Compos. Sci. Technol..

[43] Schell J., Renglli M., Lenthe G., Mueller R., Ermanni P. (2006). Micro-computed tomography determination of glass fibre rein-forced polymer mesostructure. Compos. Sci. Technol..

[44] Little J.E., Yuan X., Jones M.I. (2012). Characterisation of voids in fibre reinforced composite materials. NDT E Int..

[45] Hülsbusch D., Mrzljak S., Walther F. (2016). In situ computed tomography for the characterization of the fatigue damage development in glass fiber-reinforced polyurethane. Mater. Test..

[46] Scholz R., Delp A., Walther F. (2020). In situ characterization of damage development in Cottonid due to quasi-static tensile loading. Materials.

[47] Advani S.G., Tucker C.L. (1987). The Use of Tensors to Describe and Predict Fiber Orientation in Short Fiber Composites. J. Rheol..

[48] Ehrenstein G.W. (2006). Faserverbund-Kunststoffe: Werkstoffe, Verarbeitung, Eigenschaften.

[49] Fu S.Y., Lauke B. (1996). Effects of fiber length and fiber orientation distributions on the tensile strength of short-fiber-reinforced polymers. Compos. Sci. Technol..

[50] Laranjeira E., De Carvalho L.H., Silva S.M.D.L., D’Almeida J.R.M. (2006). Influence of Fiber Orientation on the Mechanical Properties of Polyester/Jute Composites. J. Reinf. Plast. Compos..

[51] Norman D.A., Robertson R.E. (2003). The effect of fiber orientation on the toughening of short fiber-reinforced polymers. J. Appl. Polym. Sci..

[52] Le Baillif M., Oksman K. (2009). The effect of processing on fiber dispersion, fiber length, and thermal degradation of bleached sulfite cellulose fiber polypropylene composites. J. Thermoplast. Compos. Mater..

[53] Thomason J.L., Vlug M.A. (1997). Influence of fibre length and concentration on the properties of glass fibre-reinforced polypropylene: Impact properties. Compos. Part A Appl. Sci. Manuf..

[54] Koplin T. (2014). Untersuchung der Einflussgebenden Parameter bei der Compoundierung von Cellulosefaserverstärkten Thermoplastischen Kunststoffen Mittels Eines Gleichläufigen Doppelschneckenextruders. Ph.D. Thesis.

[55] Gamon G., Evon P., Rigal L. (2013). Twin-screw extrusion impact on natural fibre morphology and material properties in poly(lactic acid) based biocomposites. Ind. Crop. Prod..

[56] Migneault S., Koubaa A., Erchiqui F., Chaala A., Englund K., Krause C., Wolcott M. (2008). Effect of fiber length on processing and properties of extruded wood-fiber/HDPE composites. J. Appl. Polym. Sci..

[57] Bledzki A.K., Faruk O., Mamun A.A. (2008). Influence of compounding processes and fibre length on the mechanical properties of abaca fibre-polypropylene composites. Polimery.

